# Effects of *EZH2* Polymorphisms on Susceptibility to and Pathological Development of Hepatocellular Carcinoma

**DOI:** 10.1371/journal.pone.0074870

**Published:** 2013-09-10

**Authors:** Yung-Luen Yu, Kuo-Jung Su, Yi-Hsien Hsieh, Hsiang-Lin Lee, Tzy-Yen Chen, Pei-Ching Hsiao, Shun-Fa Yang

**Affiliations:** 1 Graduate Institute of Cancer Biology and Center for Molecular Medicine, China Medical University, Taichung, Taiwan; 2 The Ph.D. Program for Cancer Biology and Drug Discovery, China Medical University, Taichung, Taiwan; 3 Department of Biotechnology, Asia University, Taichung, Taiwan; 4 Institute of Biochemistry and Biotechnology, School of Medicine, Chung Shan Medical University, Taichung, Taiwan; 5 Institute of Medicine, Chung Shan Medical University, Taichung, Taiwan; 6 Department of Surgery, Chung Shan Medical University Hospital, Taichung, Taiwan; 7 School of Medicine, Chung Shan Medical University, Taichung, Taiwan; 8 Department of Internal Medicine, Chung Shan Medical University Hospital, Taichung, Taiwan; 9 Department of Medical Research, Chung Shan Medical University Hospital, Taichung, Taiwan; The University of Hong Kong, China

## Abstract

**Background:**

The enhancer of zeste 2 (*EZH2*) gene encodes the histone methyltransferase that is the catalytic component of the polycomb repressive complex-2, which initiates epigenetic silencing of genes. The expression level of *EZH2* in hepatocellular carcinoma (HCC) is highly correlated with tumor progression; however, it has not been determined if specific *EZH2* genetic variants are associated with the risk of HCC. This study investigated the potential associations of *EZH2* single-nucleotide polymorphisms with HCC susceptibility and its clinicopathologic characteristics.

**Methodology/Principal Findings:**

A total of 220 HCC patients and 552 cancer-free controls were analyzed for four *EZH2* single-nucleotide polymorphisms (rs6950683, rs2302427, rs3757441, and rs41277434) using real-time PCR genotyping. After adjusting for other co-variants, the individuals carrying at least one C allele at *EZH2* rs6950683 and rs3757441 had a 0.611-fold and a 0.660-fold lower risk of developing HCC than did wild-type (TT) carriers, respectively. The CCCA or CCTA haplotype among the four *EZH2* sites (rs6950683, rs2302427, rs3757441, and rs41277434), respectively, was also associated with a reduced risk of HCC. Furthermore, HCC patients who carried at least one C allele at rs6950683 or rs3757441 had a higher lymph–node-metastasis risk but a lower liver-cirrhosis risk than did patients carrying the wild-type allele.

**Conclusions:**

The rs6950683 and rs3757441 polymorphic genotypes of *EZH2* might contribute to the prediction of susceptibility to and pathological development of HCC. This is the first study to provide insight into risk factors associated with *EZH2* variants in carcinogenesis of HCC in Taiwan.

## Introduction

Hepatocellular carcinoma (HCC) is one of the most common malignancies worldwide and the second leading cause of cancer-related death in Taiwan [1], [2]. HCC carcinogenesis is a complex multifactor and multistep process, and is associated with multiple risk factors, including chronic hepatitis B virus (HBV) or hepatitis C virus (HCV) infection, liver cirrhosis, carcinogen exposure, excessive alcohol use, and a variety of genetic factors [3]–[5].

The enhancer of zeste homolog 2 (EZH2) is a SET (Su(var)3-9, Enhancer-of-zeste and Trithorax) domain-containing methyltransferase that catalyzes the methylation of histone H3 to form the transcriptional repressive epigenetic marker H3K27me3. EZH2 is a subunit of the multi-enzyme complex polycomb repressive complex 2 and is involved in chromatin compaction and gene repression [6]. Recently, EZH2 has been linked to the aggressiveness of human cancers, including lymphomas [7], breast cancer [8], and prostate cancer [9]. Overexpression of EZH2 has been correlated with advanced stages of human cancer progression and poor prognosis [10]. In addition, EZH2 promotes epithelial–mesenchymal transition, a process that is associated with cancer progression and metastasis [11].

Epidemiological studies suggest that genetic factors, including single nucleotide polymorphisms (SNPs) are important in mediating an individual’s susceptibility to many types of cancer [12]. Several studies suggest an association between HCC risk and SNPs in certain genes. For example, specific SNPs in insulin-like growth factor 2 and 2R, plasminogen activator inhibitor, and matrix metalloproteinase 14 are HCC risk factors [13**]**–[15].

Although EZH2 contributes to the formation of many types of cancer, the association between *E* *Z* *H2* variants and HCC risk and prognosis has been poorly investigated. We, therefore, performed a case-control study of four SNPs located in the promoter, exonic, and intronic regions of *E* *Z* *H2* to assess the associations between these SNPs and HCC susceptibility and clinicopathologic characteristics.

## Materials and Methods

### Study subjects and specimen collection

This hospital-based case-control study recruited 220 HCC patients between 2007 and 2012 at the Chung Shan Medical University Hospital, Taiwan. The diagnosis of HCC was made according to the criteria specified in the national guidelines for HCC. Specifically, liver tumors were diagnosed by histology or cytology—irrespective of α-fetoprotein (AFP) titer—after computed tomography or magnetic resonance imaging data showed: (1) at least one liver mass ≥2 cm in diameter; (2) early enhancement and AFP levels ≥400 ng/ml; or (3) early arterial phase-contrast enhancement plus early venous phase-contrast washout regardless of AFP level. During the same study period, 552 ethnic group-matched individuals were enrolled as the controls that entered the physical examination at the same hospital. These control groups had neither self-reported history of cancer of any sites. Personal information and characteristics collected from the study subjects using interviewer-administered questionnaires contained questions involving demographic characteristics and the status of cigarette smoking and alcohol drinking. HCC patients were clinically staged at the time of diagnosis according to the tumor, node, metastasis (TNM) staging system of the American Joint Committee on Cancer (2002) [16]. Liver cirrhosis was diagnosed by liver biopsy, abdominal sonography, or biochemical evidence of liver parenchymal damage with endoscopic esophageal or gastric varices. The patients’ clinicopathological characteristics, including clinical staging, tumor size, lymph-node metastasis, distant metastasis, presence of HBV surface antigen (HBsAg), reactivity with antibody against HCV (anti-HCV), liver cirrhosis, AFP, aspartate aminotransferase (AST) and alanine aminotransferase (ALT) levels, were verified by chart review. Whole blood specimens collected from the controls and HCC patients were placed in tubes containing ethylenediaminetetraacetic acid (EDTA), immediately centrifuged, and stored at −80°C. Before commencing the study, approval was obtained from the Institutional Review Board of Chung Shan Medical University Hospital and informed written consent was obtained from each individual.

### Selection of *EZH2* Polymorphisms

A total of four single nucleotide polymorphisms (SNPs) of the EZH2 gene (NM_004456) were selected from the International HapMap Project data for this study. We included the non-synonymous SNPs rs2302427 (D185H in exon 6) in the coding sequences of the gene. Furthermore, others SNPs (rs6950683, rs3757441 and rs41277434) were selected in this study since these SNPs were found in the cancer patients [17], [18].

### Genomic DNA extraction

Genomic DNA was extracted using QIAamp DNA blood mini kit reagents (Qiagen, Valencia, CA). DNA was dissolved in TE buffer [10 mM Tris (pH 7.8), 1 mM EDTA] and then quantified by measurement of its solution’s optical density at 260 nm. Final DNA preparations were stored at −20°C and used as templates for polymerase chain reaction (PCR).

### Real-time PCR

Allelic discrimination of the *E* *Z* *H2* rs6950683, rs2302427, rs3757441, and rs41277434 gene polymorphisms was assessed using an ABI StepOne™ Real-Time PCR System (Applied Biosystems), SDS v3.0 software (Applied Biosystems), and the TaqMan assay. The final volume for each reaction mixture was 5 µL, containing 2.5 µL TaqMan Genotyping Master Mix, 0.125 µL TaqMan probes mix, and 10 ng genomic DNA. The reaction conditions included an initial denaturation step at 95°C for 10 min followed by 40 cycles at 95°C for 15 sec and 60°C for 1 min. For each assay, appropriate controls (nontemplate and known genotype) were included in each typing run to monitor reagent contamination and as a quality control. To validate results from real-time PCR, around 5% of assays were repeated and several cases of each genotype were confirmed by the DNA sequence analysis.

### Statistical analysis

Hardy–Weinberg equilibrium was assessed using a chi-square goodness-of-fit test for biallelic markers. A Mann–Whitney U-test and a Fisher’s exact test were used to compare differences of age and demographic characteristics distributions between controls and HCC patients. The odds ratios (ORs) with 95% confidence intervals (CIs) were estimated by logistic regression models. The adjusted odds ratios (AORs) with 95% CIs of the association between genotype frequencies and HCC risk as well as clinical pathological characteristics were estimated by multiple logistic regression models after controlling for other covariates. The haplotype-based analysis was using the Phase program. All *p* values <0.05 were considered significant. The data were analyzed using SAS statistical software (Version 9.1, 2005; SAS Institute Inc., Cary, NC).

## Results

We found that 37.5% of the healthy control subjects (207 of 552) and 35% of the patients with HCC (77 of 220), consumed alcohol; 39.1% of the healthy controls (216 of 552) and 39.1% of the patients with HCC (86 of 220) smoked tobacco. No significant differences were found in the distribution of alcohol consumption (*p* = 0.561) and tobacco use (*p* = 0.992) between healthy controls and patients with HCC, whereas the age (control: 51.65±14.62; HCC: 64.50±11.90; *p* < 0.001) and gender (*p* = 0.001) distributions between the two groups were significantly different (Table 1). To reduce possible interference of confounding variables, AORs with 95% CIs were estimated by multiple logistic regression models after controlling for age and gender in each comparison. Table 2 shows the genotype distributions and the association between HCC and *E* *Z* *H2* polymorphisms. In our recruited control group, the frequencies of *E* *Z* *H2* rs6950683 (χ^2^ value: 5.59), rs2302427 (χ^2^ value: 5.77), rs3757441 (χ^2^ value: 1.79), and rs41277434 (χ^2^ value: 0.32) were in Hardy-Weinberg equilibrium, respectively. The alleles with the highest distribution frequency at *E* *Z* *H2* rs6950683, rs2302427, rs3757441, and rs41277434 in both HCC patients and controls were homozygous T/T, homozygous C/C, homozygous T/T, and homozygous A/A, respectively. Individuals carrying CC or TC + CC at rs6950683 showed a 0.288-fold (95% CI: 0.130–0.638) and a 0.611-fold (95% CI: 0.419–0.891) lower risk of HCC, and those carrying CC or TC + CC at rs3757441 showed a 0.273-fold (95% CI: 0.116–0.645) and a 0.660-fold (95%CI: 0.453–0.962) lower risk of HCC compared with individuals carrying the wild-type allele. Individuals with polymorphisms at rs2302427 and rs41277434 showed no reduction in HCC risk compared with wild-type individuals.

**Table 1:** Demographic characteristics of controls and patients with HCC.

Variable	Controls (N = 552)	Patients (N = 220)	*p* value
**Age (yrs)**	**Mean ± S.D.**	**Mean ± S.D.**	
	51.65±14.62	64.50±11.90	<0.001*
**Gender**	(%)	(%)	
Male	449 (81.3%)	154 (70.0%)	
Female	103 (18.7%)	66 (30.0%)	0.001*
**Alcohol consumption**			
No	345 (62.5%)	143 (65.0%)	
Yes	207 (37.5%)	77 (35.0%)	0.561
**Tobacco use**			
No	336 (60.9%)	134 (60.9%)	
Yes	216 (39.1%)	86 (39.1%)	0.992
**Stage**			
I		84 (38.2%)	
II		58 (26.4%)	
III		65 (29.5%)	
IV		13 (5.9%)	
**Tumor T status**			
≤T2		145 (65.9%)	
>T2		75 (34.1%)	
**Lymph node status**			
N0		211 (95.9%)	
N1 + N2		9 (4.1%)	
**Metastasis**			
M0		209 (95.0%)	
M1		11 (5.0%)	

* , considered statistically significant.

S.D., standard deviation.

**Table 2:** Distribution of *EZH2* genotypes in healthy controls and patients with HCC.

Variable	Controls (N = 552) (%)	Patients (N = 220) (%)	OR (95% CI)	AOR (95% CI)
**rs6950683**				
TT	264 (47.8%)	132 (60.0%)	1.00	1.00
TC	220 (39.9%)	77 (35.0%)	0.700 (0.502–0.977)*	0.711 (0.478–1.056)
CC	68 (12.3%)	11 (5.0%)	0.324 (0.165–0.632)*	0.288 (0.130–0.638)*
TC + CC	288 (52.2%)	88 (40.0%)	0.611 (0.445–0.839)*	0.611 (0.419–0.891)*
**rs2302427**				
CC	346 (62.7%)	135 (61.4%)	1.00	1.00
CG	171 (31.0%)	75 (34.1%)	1.124 (0.803–1.574)	1.086 (0.723–1.630)
GG	35 (6.3%)	10 (4.5%)	0.732 (0.353–1.520)	0.480 (0.211–1.093)
CG + GG	206 (37.3%)	85 (38.6%)	1.058 (0.767–1.459)	0.944 (0.644–1.383)
**rs3757441**				
TT	271 (49.1%)	131 (59.5%)	1.00	1.00
TC	223 (40.4%)	80 (36.4%)	0.742 (0.534–1.032)	0.771 (0.520–1.144)
CC	58 (10.5%)	9 (4.1%)	0.321 (0.154–0.668)*	0.273 (0.116–0.645)*
TC + CC	281 (50.9%)	89 (40.5%)	0.655 (0.477–0.899)*	0.660 (0.453–0.962)*
**rs41277434**				
AA	517 (93.6%)	209 (95.0%)	1.00	1.00
AC	34 (6.2%)	11 (5.0%)	0.800 (0.398–1.609)	0.765 (0.350–1.670)
CC	1 (0.2%)	0 (0%)	----	----
AC + CC	35 (6.4%)	11 (5.0%)	0.777 (0.388–1.560)	0.727 (0.334–1.585)

AORs with their 95% CIs were estimated by multiple logistic regression models after controlling for age and gender. *, considered statistically significant.

The distribution of clinical status and *E* *Z* *H2* genotypes in HCC patients were estimated to clarify the role of *E* *Z* *H2* polymorphisms in the clinicopathologic state of HCC patients. Clinical status assessments included TNM staging, primary tumor size, lymph node involvement, distant metastasis, presence of HBV or HCV, and liver cirrhosis. Compared with the wild-type genotype, patients with at least one polymorphic C allele at *E* *Z* *H2* rs6950683 (Table 3) or rs3757441 (Table 4) showed a 19.029-fold (95% CI: 1.733–208.866) or a 19.067-fold (95% CI: 1.747–208.155) increase in lymph-node metastasis, but a 0.421-fold (95% CI: 0.182–0.973) or a 0.481-fold (95% CI: 0.209–1.110) decrease in liver cirrhosis, respectively. No significant differences were observed between other *E* *Z* *H2* genotypic frequencies and any clinicopathological variable (data not shown).

**Table 3:** Associations of clinical status and *EZH2* rs6950683 genotypic frequencies in 220 HCC patients.

Variable	Genotypic frequencies			
	TT (N = 132)	TC + CC (N = 88)	OR (95% CI)	AOR (95% CI)
	(%)	(%)		
**Clinical Stage**				
Stage I/II	88 (66.7%)	54 (61.4%)	1.00	1.00
Stage III/IV	44 (33.3%)	34 (38.6%)	1.259 (0.718–2.207)	1.018 (0.472–2.197)
**Tumor size**				
≤T2	89 (67.4%)	56 (63.6%)	1.00	1.00
>T2	43 (32.6%)	32 (36.4%)	1.183 (0.671–2.084)	0.894 (0.409–1.955)
**Lymph node metastasis**				
No	130 (98.5%)	81 (92.0%)	1.00	1.00
Yes	2 (1.5%)	7 (8.0%)	5.617 (1.139–27.705)*	19.027 (1.733–208.866)*
**Distant metastasis**				
No	126 (95.5%)	83 (94.3%)	1.00	1.00
Yes	6 (4.5%)	5 (5.7%)	1.265 (0.374–4.280)	3.198 (0.482–21.216)
**Child-Pugh grade**				
A	94 (71.2%)	69 (78.4%)	1.00	1.00
B or C	38 (28.8%)	19 (21.6%)	0.681 (0.362–1.282)	0.502 (0.217–1.162)
**HBsAg**				
Negative	73 (55.3%)	56 (63.6%)	1.00	1.00
Positive	59 (44.7%)	32 (36.4%)	0.707 (0.406–1.230)	0.9452 (0.196–1.032)
**Anti-HCV**				
Negative	70 (53.0%)	43 (48.9%)	1.00	1.00
Positive	62 (47.0%)	45 (51.1%)	1.182 (0.689–2.027)	1.896 (0.918–3.919)
**Liver cirrhosis**				
Negative	26 (19.7%)	30 (34.1%)	1.00	1.00
Positive	106 (80.3%)	58 (65.9%)	0.474 (0.256–0.877)*	0.421 (0.182–0.973)*

AORs with their 95% CIs were estimated by multiple logistic regression models after controlling for age, gender, tobacco use and alcohol consumption.

>T2: multiple tumor >5 cm in the greatest dimension or tumor involving a major branch of the portal or hepatic vein(s)

* , considered statistically significant.

**Table 4:** Associations of clinical status and *EZH2* rs3757441 genotypic frequencies in 220 HCC patients.

Variable	Genotypic frequencies			
	TT (N = 131)	TC + CC (N = 89)	OR (95% CI)	AOR (95% CI)
	(%)	(%)		
**Clinical Stage**				
Stage I/II	88 (67.2%)	54 (60.7%)	1.00	1.00
Stage III/IV	43 (32.8%)	35 (39.3%)	1.326 (0.757–2.323)	1.365 (0.635–2.936)
**Tumor size**				
≤T2	90 (68.7%)	55 (61.8%)	1.00	1.00
>T2	41 (31.3%)	34 (38.2%)	1.357 (0.771–2.388)	1.395 (0.640–3.039)
**Lymph node metastasis**				
No	129 (98.5%)	82 (92.1%)	1.00	1.00
Yes	2 (1.5%)	7 (7.9%)	5.506 (1.116–27.154)*	19.067 (1.747–208.155)*
**Distant metastasis**				
No	125 (95.4%)	84 (94.4%)	1.00	1.00
Yes	6 (4.6%)	5 (5.6%)	1.240 (0.367–4.195)	2.994 (0.445–20.140)
**Child-Pugh grade**				
A	97 (74.0%)	66 (74.2%)	1.00	1.00
B or C	34 (26.0%)	23 (25.8%)	0.994 (0.538–1.838)	0.702 (0.312–1.582)
**HBsAg**				
Negative	73 (55.7%)	56 (62.9%)	1.00	1.00
Positive	58 (44.3%)	33 (37.1%)	0.742 (0.427–1.287)	0.442 (0.201–1.038)
**Anti-HCV**				
Negative	70 (53.4%)	43 (48.3%)	1.00	1.00
Positive	61 (46.6%)	46 (51.7%)	1.228 (0.716–2.105)	2.055 (0.993–4.255)
**Liver cirrhosis**				
Negative	28 (21.4%)	28 (31.5%)	1.00	1.00
Positive	103 (78.6%)	61 (68.5%)	0.592 (0.321–1.092)	0.481 (0.209–1.110)

The AORs with their 95% CI were estimated by multiple logistic regression models, after controlling for age, gender, tobacco use and alcohol consumption.

>T2: multiple tumor more than 5 cm or tumor involving a major branch of the portal or hepatic vein(s)

* , considered statistically significant.

AFP, AST, and ALT are common clinical pathological markers of HCC. To clarify the relationship between clinical status and the levels of these markers in HCC patients, we analyzed the association of these pathological markers with *E* *Z* *H2* genotypic frequencies. No significant association was found between the levels of these HCC clinical pathological markers and genotypes for any of the *E* *Z* *H2* SNPs in HCC patients (Table 5).

**Table 5:** Association of *EZH2* genotypic frequencies with HCC blood biochemistry results.

Characteristic	α-Fetoprotein (ng/ml)	AST (IU/l)	ALT (IU/l)	AST/ALT ratio
**rs6950683**				
TT	2864.8±1153.3	172.1±30.7	152.5±27.0	1.49±0.12
TC/CC	5594.1±2106.9	182.6±37.6	142.7±23.1	1.51±0.12
*p* value	0.221	0.829	0.797	0.935
**rs2302427**				
CC	4461.0±1664.7	184.9±28.5	141.5±17.6	1.60±0.13
CG/GG	3155.3±1001.1	162.5±41.7	159.7±39.3	1.34±0.08
*p* value	0.561	0.647	0.636	0.133
**rs3757441**				
TT	2842.4±1162.1	176.7±31.4	159.4±27.9	1.46±0.12
TC/CC	5580.2±2083.0	175.6±36.4	132.6±20.9	1.56±0.13
*p* value	0.221	0.981	0.480	0.541
**rs41277434**				
AA	4161.0±1147.1	182.0±24.9	153.2±19.5	1.51±0.09
AC/CC	72.61±38.5	55.2±16.6	60.9±16.9	1.27±0.18
*p* value	0.415	0.293	0.281	0.542

Mann-Whitney U test was used between two groups.

Values presented are the mean ± standard error.

The haplotype distributions of *E* *Z* *H2* rs6950683, rs2302427, rs3757441, and rs41277434 were further evaluated and seven haplotypes were derived from these four SNPs in our recruited individuals. The most common haplotype in the control group was TCTA (42.4%), and it was, therefore, chosen as the reference. Compared with this reference, two minor haplotypes CCCA and CCTA significantly reduced the risk of HCC by 0.573-fold (95% CI: 0.435–0.755) and 0.200-fold (95% CI: 0.046–0.863), respectively (Table 6).

**Table 6:** Distribution frequency of *EZH2* haplotype in control and HCC patients.

Variable				Controls (N = 1104) (%)	Patients (N = 440) (%)	OR (95% CI)	*p* value
rs6950683 T/C	rs2302427C/G	rs3757441T/C	rs41277434 A/C				
T	C	T	A	468 (42.4%)	234 (53.2%)	Reference	
C	C	C	A	335 (30.3%)	96 (21.8%)	0.573 (0.435–0.755)	< 0.001*
T	G	T	A	240 (21.7%)	94 (21.4%)	0.783 (0.589–1.042)	0.093
T	C	T	C	36 (3.3%)	11 (2.5%)	0.611 (0.306–1.222)	0.160
C	C	T	A	20 (1.8%)	2 (0.5%)	0.200 (0.046–0.863)	0.017*
T	C	C	A	4 (0.4%)	2 (0.5%)	1.000 (0.182–5.499)	1.000
C	G	T	A	1 (0.1%)	1 (0.2%)	1.665(0.151–18.425)	0.674

* , considered statistically significant.

## Discussion

The major etiologies for HCC in Taiwan include infection with HBV or HCV, alcohol consumption, history of liver cirrhosis, and family history of HCC [15], [19], [20]. In our HCC group, however, alcohol consumption and tobacco use were not significantly different from those of healthy controls (Table 1), suggesting that these two risk factors alone do not fully explain the pathogenesis of HCC and that genetic components may play a pivotal role. This is consistent with the observations that many gene polymorphisms and somatic mutations have been associated with the preneoplastic stage of HCC [15], [21], [22].

EZH2 plays an important role in cell-cycle regulation, and its gene has emerged as a novel oncogene and putative oncological therapy target [23]. Therefore, *E* *Z* *H2* polymorphisms may be associated with the development of HCC. *E* *Z* *H2* contains 20 exons, 19 introns, and 41 identified SNPs [24], and encodes two isoforms of different transcript size [18]. In this hospital-based case-control study, four *E* *Z* *H2* SNPs were genotyped in 220 patients with HCC and 552 healthy controls. We observed that at least one polymorphic C allele at SNPs rs6950683 and rs3757441 is strongly associated with reduced HCC risk (Table 2). Rs3757441 is an intronic SNP, and as such may affect gene expression through several mechanisms, including changes in transcription–factor binding sites [25], microRNA-targeting sequences [26], and splicing variants [27]. Rs6950683, is located upstream of exon 1, and, therefore, may impact gene expression by affecting promoter function. Further functional studies are needed to confirm the specific mechanisms by which these *E* *Z* *H2* polymorphisms influence the development of HCC.

Although the functional importance of rs6950683 and rs3757441 has not been tested experimentally, it has been observed that individuals carrying C/C alleles at these two SNPs have a lower risk of lung cancer than do those carrying the T/T wild-type allele [24]. This study provides novel information on the effects of single nucleotide polymorphisms in *E* *Z* *H2* on HCC susceptibility and clinicopathology, but found that HCC patients carrying rs6950683 and rs3757441 polymorphisms have a higher risk of lymph node metastasis than wild-type carriers. However, the number of individuals examined in this study was relatively small, and additional studies with more patients are needed to verify the effects of *E* *Z* *H2* polymorphisms on HCC that we observed and to explore the effects of these variants on the biological function of *E* *Z* *H2*.

Although many SNPs have no direct effect on gene products, they can still be used as genetic markers to locate adjacent functional variants that contribute to disease. In addition, the contribution of SNPs to a disease-related haplotype may not be apparent when looking at individual SNPs. Therefore, haplotype analysis is sometimes advantageous over analysis of individual SNPs for detecting an association between alleles and a disease phenotype [28]. Our haplotype analysis of the four *E* *Z* *H2* SNPs rs6950683, rs2302427, rs3757441, and rs41277434 revealed that the CCCA and CCTA haplotypes are associated with a lower risk of HCC (Table 6). However, it is possible that these *E* *Z* *H2* SNPs are linked with other functional polymorphisms and are, therefore, not directly responsible for the decreased susceptibility to HCC.

In conclusion, this is the first study to show a significant association between polymorphisms in *E* *Z* *H2* and HCC risk. These findings suggest that the presence of a variant *E* *Z* *H2* allele may be a protective factor for the development of HCC and could be a useful genetic marker for predicting susceptibility to HCC.
